# *In vitro* labeling of endothelial progenitor cells isolated from peripheral blood with superparamagnetic iron oxide nanoparticles

**DOI:** 10.3892/mmr.2012.912

**Published:** 2012-05-10

**Authors:** JUN-HUI SUN, YUE-LIN ZHANG, CHUN-HUI NIE, SU-PING QIAN, XIAO-BO YU, HAI-YANG XIE, LIN ZHOU, SHU-SEN ZHENG

**Affiliations:** 1 Key Laboratory of Combined Multi-Organ Transplantation, Ministry of Public Health, Key Laboratory of Organ Transplantation, and Division of Hepatobiliary and Pancreatic Surgery, Department of Surgery, First Affiliated Hospital, School of Medicine, Hangzhou, Zhejiang, P.R. China; 2 Molecular Imaging Platform, Zhejiang-California International Nanosystems Institute, Zhejiang University, Hangzhou, Zhejiang, P.R. China

**Keywords:** endothelial progenitor cell, superparamagnetic iron oxide nanoparticles, cell labeling

## Abstract

The transplantation of endothelial progenitor cells (EPCs) provides a novel method for the treatment of human tumors or vascular diseases. Magnetic resonance imaging (MRI) has proven to be effective in tracking transplanted stem cells by labeling the cells with superparamagnetic iron oxide (SPIO) nanoparticles. The SPIO has been used to label and track the EPCs; however, the effect of SPIO upon EPCs remains unclear on a cellular level. In the present study, EPCs were labeled with home-synthesized SPIO nanoparticles *in vitro* and the biological characteristics of the labeled EPCs were evaluated. The EPCs were isolated from the peripheral blood of New Zealand rabbits and cultured in fibronectin-coated culture flasks. The EPCs were labeled with home-synthesized SPIO nanoparticles at a final iron concentration of 20 μg/ml. Labeled EPCs were confirmed with transmission electron microscopy and Prussian blue staining. The quantity of iron/cell was detected by atomic absorption spectrometry. The membranous antigens of EPCs were detected by cytofluorimetric analysis. Cell viability and proliferative capability between the labeled and unlabeled EPCs were compared. The rabbit EPCs were effectively labeled and the labeling efficiency was approximately 95%. The SPIO nanoparticles were localized in the endosomal vesicles of the EPCs, which were confirmed by transmission electron microscopy. No significant differences were found in cell viability and proliferative capability between labeled and unlabeled EPCs (P>0.05). In conclusion, rabbit peripheral blood EPCs were effectively labeled by home-synthesized SPIO nanoparticles, without influencing their main biological characteristics.

## Introduction

As a potential method, cell transplantation provides a novel strategy for the therapy of incurable human diseases (1–3). Endothelial progenitor cells (EPCs) isolated from peripheral blood play a significant role in the treatment of injured blood vessels and ischemic tissues. Numerous studies have shown that EPCs exist in the peripheral blood and migrate into the damaged endothelium and neovascularization sites. They may play a role in the therapeutic procedure of repairing the endothelium and in angiogenesis (4,5). Furthermore, EPCs are capable of migrating into the tumor and participating in tumor angiogenesis (6,7).

In order to better understand the mechanism of EPC therapy, the *in vivo* monitoring of the cellular dynamics of transplanted EPCs has been proposed. A non-invasive *in vivo* technique that permits an evaluation of the potential migration of the transplanted EPCs would prove to be an essential tool for the treatment procedure. Many studies have indicated that magnetic resonance imaging (MRI) is effective in tracking the distribution of transplanted EPCs *in vivo* by labeling the cells with superparamagnetic iron oxide (SPIO) nanoparticles (8).

However, it is not very clear whether the SPIO labeling technique in EPCs is effective and safe. Additionally, the effect of SPIO upon EPCs remains unclear on a cellular level (9). The objective of the present study was to investigate whether and to what extent the labeling of EPCs with SPIO affects the main biological characteristics of EPCs.

## Materials and methods

### EPC culture and characterization

The present study was approved by the Animal Use and Care Committee of our institution. EPCs were generated from the peripheral blood of 5 adult New Zealand white rabbits weighing 2–2.5 kg. Blood (20 ml) was obtained from the central ear artery of the rabbits. The fresh blood was heparinized and then diluted with phosphate-buffered saline (PBS), and the layer of peripheral blood mononuclear cells was selected with density centrifugation and was then resuspended in microvascular growth medium-2 (EGM-2 MV; Cambrex, Walkersville, MD, USA) supplemented with 10% fetal bovine serum (SAFC Biosciences, St. Louis, MO, USA). Before being planted in a 25-cm^3^ culture flask, the cells were gently blown in the culture solution, adjusted to 1×10^6^/ml of the concentration, and then grown in standard culture medium at 37°C with 5% CO_2_. The growth and morphology of EPCs in the culture was observed every day with an inverted phase-contrast microscope (Axioscop; Zeiss Co. Ltd., Oberkochen, Germany).

In order to characterize the EPCs, the expression of membranous antigen on the cells cultured after 7 days was detected by cytofluorimetric analysis with a flow cytometer (Becton-Dickinson, San Jose, CA, USA). The primary anti-human antibodies (cross-reaction with rabbit), anti-CD31, anti-CD34 and anti-CD133 (BioLegend, San Diego, CA, USA), were employed.

### EPC labeling and identification of the labeled EPCs

The SPIO (Fe_3_O_4_) nanoparticles were presented by S.P. Q. (Molecular Imaging Platform, Zhejiang-California International Nanosystems Institute, Zhejiang University, Hangzhou, China). The EPCs were grown in 25-cm^3^ flasks. After 21 days, the grown cells were transferred to the culture medium containing SPIO nanoparticles for labeling. The concentration of 20 μg/ml iron was used for culture. The EPCs were incubated continuously for 24 h at 37°C in a 95% air/5% CO_2_ incubator.

The EPCs were collected by removing the free SPIO nanoparticles and washed with PBS 3 times. For the purpose of Prussian blue staining to identify the EPC profile and intracellular iron nanoparticles, the cells were continuously incubated for 20 min with 2% potassium ferrocyanide in 6% hydrochloric acid, and then counterstained with nuclear fast red for 3 min.

To detect the iron concentration within the EPCs, the EPC suspension was dissolved in 37% hydrochloric acid followed by analysis with a polarized atomic absorption spectrometer (Shengyang Huaguang HG-9602A, Shengyang, China). The analysis process was repeated 3 times and the mean value was obtained. The distribution of the SPIO nanoparticles within the EPCs was shown under an electron microscope. The harvested labeled EPCs were fixed at 4°C in 2.5% buffered glutaraldehyde for 1 h, followed by 1% osmium tetroxide for 2 h. The samples of these EPCs were examined with a transmission electron microscope (H600; Hitachi, Tokyo, Japan).

### Labeled EPC viability and proliferation

Cell viability and proliferative activity of SPIO-labeled and -unlabeled EPCs were evaluated and compared. All procedures were performed 3 times.

EPC viability was evaluated by trypan blue staining. The proliferative activity of the EPCs was observed under a light microscope (Axioscop; Zeiss). Additionally, tetrazolium salt (MTT) assay was performed to evaluate the toxicity and the effect of SPIO labeling upon EPC proliferation. The EPCs of passage (P)_1_ were grown in 96-well plates at 1×10^4^ cells/well. SPIO solution at a final iron concentration of 20 μg/ml was added into 40 wells, and the remaining 40 wells to which SPIO was not added served as the control. The absorbance values of the unlabeled EPCs and SPIO-labeled EPCs were measured from days 1 to 5 of the culture process (8 wells/day). For the assay, 20 μl of MTT (5 mg/ml; Fluka Co., St. Gallen, Switzerland) were added into each well and incubated at 37°C in 5% CO_2_ for 4 h. Dimethyl sulfoxide (DMSO; 150 μl; Sigma-Aldrich, St. Louis, MO, USA) was added and the medium was stirred for 10 min. When the indigo crystals (formazan crystals) were dissolved evenly in the medium, the light absorption value of each well was measured with a spectrophotometer (Model 680; Bio-Rad Laboratories, Inc., Hercules, CA, USA) using a 490-nm wavelength.

### Statistical analyses

Statistical analyses were performed using the SPSS^®^ statistical package, version 11.0 (SPSS Inc., Chicago, IL, USA) for Windows^®^. Data are presented as the means ± standard deviation (SD). To compare the differences between the labeled and unlabeled EPCs in the various experiments, the Kruskal-Wallis rank sum test was used to calculate the difference in absorbance of MTT. A p-value (two-tailed) <0.05 denoted a statistically significant difference.

## Results

### EPC morphology and characterization

In the present study, EPCs were obtained from rabbit peripheral blood and purified by density centrifugation.

**Figure 1:**
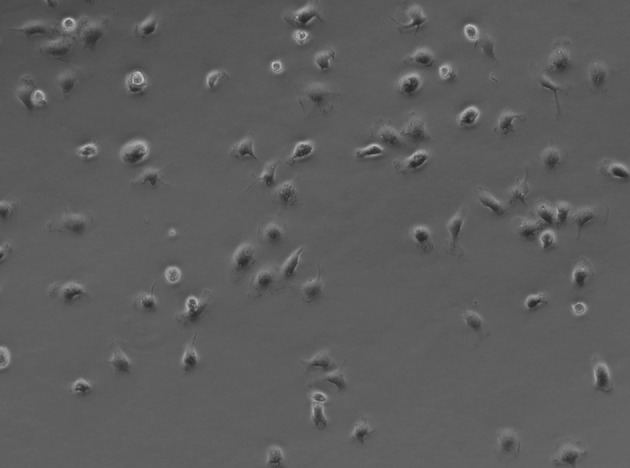
Under a light microscope, the endothelial progenitor cells (EPCs) after isolation and culture at day 3 had a round shape (magnification, ×20).

**Figure 2:**
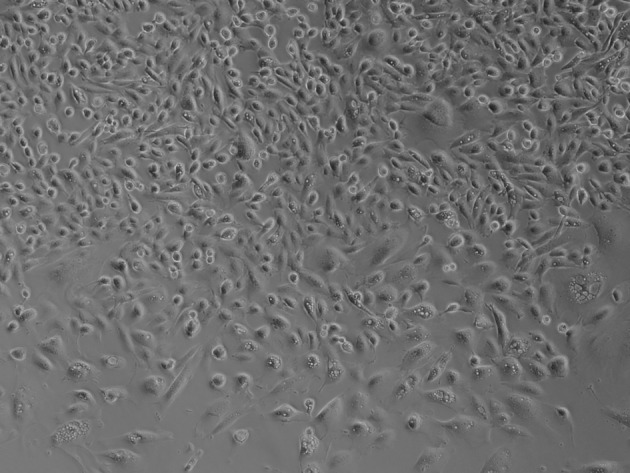
On the 7th day, the endothelial progenitor cells (EPCs) had a spindle shape and cell clusters had formed (magnification, ×20).

**Figure 3:**
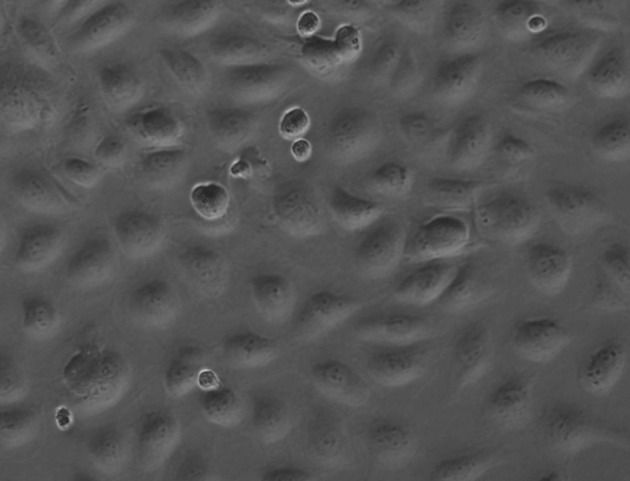
At day 21 after culture, the endothelial progenitor cells (EPCs) showed an endothelium-like cobblestone morphology (magnification, ×100).

The morphology of the obtained EPCs was similar to that described in the literature (10). During culture, inverse microscopy of EPCs showed that isolated EPCs had a round shape with variable sizes (Fig. 1). After 1 week in culture, the cells became spindle-shaped, with a centrally located nucleus, and sometimes formed cluster-like colonies (Fig. 2). The cells grew and divided rapidly, and tended to touch each other. After 3 weeks, the cultured EPCs showed the typical ‘cobblestone’ morphology of endothelial cells when they grow in colonies (Fig. 3). When they were passed to P_6_, the homogeneity of the cells reached approximately 99%.

**Figure 4:**
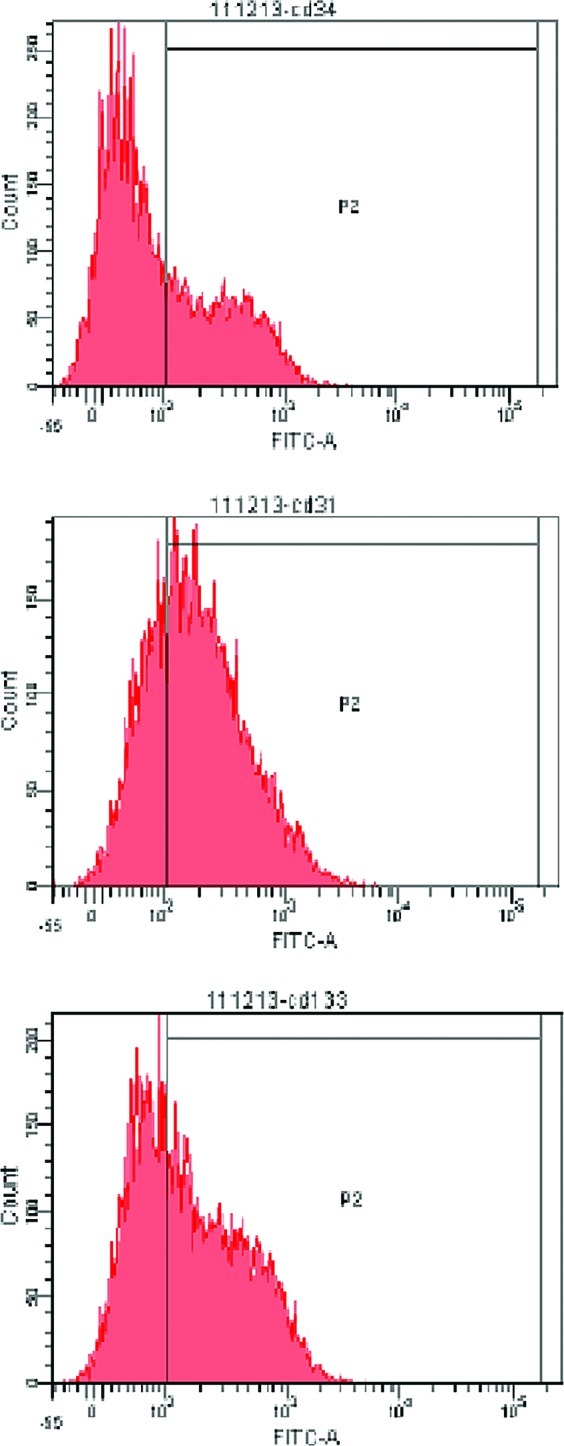
On the 7th day of culture, flow cytometric analysis showed that the cells were positive for endothelial progenitor cell (EPC)-related markers, CD31 (36.3%), CD34 (68.4%) and CD133 (56.0%).

The expression of surface markers, including CD34, CD31 and CD133, is typically found on EPCs as a demonstration of their immature character. The flowcytometry analyses revealed that 36.3±1.2, 68.4±2.3 and 56.0±2.5% of the cultured cells were positive for CD34, CD31 and CD133, respectively, after being cultured for 7 days (Fig. 4).

### EPC labeling and identification of the labeled EPCs

**Figure 5:**
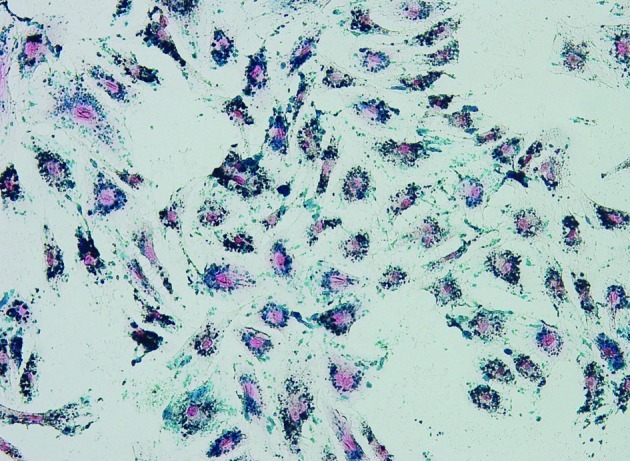
Prussian blue staining showing that the intracytoplasmic blue particles are clearly visible in most endothelial progenitor cells (EPCs). The labeled rate reached approximately 95% (magnification, ×100).

The prevalence of iron content in the labeled EPCs was revealed by Prussian blue staining and transmission electron microscopy. Cells were stained with Prussian blue, and blue iron particles were found within the labeled EPCs (Fig. 5), while no blue iron particles were found in unlabeled EPCs. Microscopic cell counting post Prussian blue staining showed that the SPIO labeling rate was >95% among all the EPCs.

**Figure 6:**
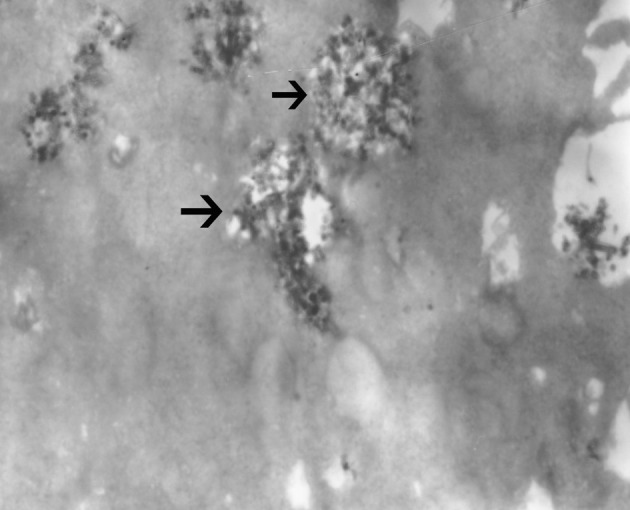
Transmission electron micrograph showing superparamagnetic iron oxide (SPIO) nanoparticles in black clusters (arrowhead) located in the endosomal vesicles outside the endothelial progenitor cell (EPC) nucleus (magnification, ×15,000).

The iron quantification per cell measured by an atomic absorption spectrometer was 13.6±1.8 pg. Transmission electron microscopy showed SPIO nanoparticles located in the endosomal vesicles in the cytoplasm of labeled EPCs (Fig. 6).

### Labeled EPC viability and proliferative capability

Microscopic cell counting after trypan blue exclusion testing revealed a mean viability of 97.5±2.1% for the SPIO-labeled EPCs. The mean viability of the unlabeled EPCs was 96.3±2.9%. There were no significant differences in viability between the labeled and unlabeled EPCs (p>0.05). The characteristics of the labeled EPCs, including figure, shape and nucleolus structure, did not differ from those of the unlabeled EPCs under a light microscope.

From days 1 to 5, the absorbence of the labeled EPCs was 0.196±0.005, 0.205±0.011, 0.244±0.013, 0.309±0.014 and 0.363±0.022, respectively. The absorbence of the unlabeled EPCs was 0.190±0.007, 0.202±0.010, 0.249±0.015, 0.315±0.016 and 0.377±0.019, respectively. There were no significant differences in MTT absorbance values between the labeled and unlabeled EPCs at each time-point (p>0.05).

## Discussion

The transplantation of stem cells is a potential strategy for the treatment of many types of human diseases due to its capability of regenerating tissues and organs. Cell transplantation has the advantages of lower cost and risk compared to organ transplantation (11,12). Furthermore, autologous cell transplantation has no risk of immunological rejection. Among different types of cells, ESCs have been previously demonstrated to have better characteristics in terms of applicability for transplantation; studies have indicated that ESCs are capable of multi-directional differentiation (13,14). EPCs were first isolated from human peripheral blood by Asahara *et al* (15). Over the past decade, the plasticity of EPCs has been intensively investigated. Many studies have demonstrated the great therapeutic potential of EPCs in tissue repair and wound healing (16–18). There are also less ethical and social controversies associated with the isolation of EPCs from bone marrow and peripheral blood, and the use of EPCs isolated from peripheral blood offers several advantages, such as easy collection and rapid *in vivo* and *in vitro* repopulation. It has been concluded by many studies that EPCs promote tissue vascular regeneration *in vivo* and provide potential treatments for ischemia, wound healing, vascular insufficiency and tumor inhibition (19). Moreover, EPCs play an essential role in post-natal neovascularization and maintaining angiogenesis (18,20). Additionally, certain evidence suggests that EPCs have a potentially protective role in endothelial dysfunction in early atherosclerosis formation (21,22).

For a better understanding of the destiny of transplanted cells following transplantation, it is essential to monitor their migration and differentiation. In order to monitor the transplanted cells, several non-invasive *in vivo* tracking imaging techniques, such as MRI, nuclear medicine and optical imaging, have been investigated (23–25). The MRI technique holds obvious advantages, as it has a wide variety of imaging sequences, high resolution and better soft-tissue contrast without radiation damage. The labeled cells are easily detected by MRI using a cell labeling technique with specific agents. Certain evidence implies that SPIO nanoparticles have strong penetrating capabilities among the MRI tracing agents, which makes it possible to cause signal change in MRI at a low-tracer concentration (26). A number of studies have already revealed the feasibility of *in vivo* tracking of the transplanted stem cells by labeling them with SPIO nanoparticles (27–29). Studies have also reported that mononuclear cells isolated from peripheral blood were tracked with MRI using colloidal superparamagnetic nanoparticles (30,31). Evidently, the low efficiency of loading these particles into the cells and the cytotoxicity of these particles limit their usage as the tracing probe (32). It has been concluded by a number of studies that the SPIO nanoparticles have little toxicity and few side-effects for cell biological characteristics (32,33). However, few studies have investigated the biological effect of SPIO upon labeled EPCs on a cellular level. Therefore, it is important to discover an efficient labeling method without deleterious effects on EPC viability and proliferative capability.

In the present study, we show that EPCs from peripheral blood of rabbits can be effectively labeled by home-synthesized SPIO nanoparticles. Our results demonstrated that there were no differences in cell viability and proliferative capability between the SPIO-labeled EPCs and unlabeled EPCs. Therefore, labeled home-synthesized SPIO nanoparticles have little influence on the main biological properties of EPCs. Additionally, these results are important for the application of MRI to localize and monitor the transplanted magnetically labeled EPCs by *in vivo* techniques.

## References

[1] Orlic D, Kajstura J, Chimenti S (2001). Mobilized bone marrow cells repair the infarcted heart, improving function and survival. Proc Natl Acad Sci USA.

[2] Sato Y, Araki H, Kato J (2005). Human mesenchymal stem cells xenografted directly to rat liver are differentiated into human hepatocytes without fusion. Blood.

[3] Qian H, Yang H, Xu W (2008). Bone marrow mesenchymal stem cells ameliorate rat acute renal failure by differentiation into renal tubular epithelial-like cells. Int J Mol Med.

[4] Murasawa S, Asahara T (2005). Endothelial progenitor cells for vasculogenesis. Physiology.

[5] Schmidt-Lucke C, Rössig L, Fichtlscherer S (2005). Reduced number of circulating endothelial progenitor cells predicts future cardiovascular events: proof of concept for the clinical importance of endogenous vascular repair. Circulation.

[6] Liang PH, Tian F, Lu Y (2011). Vascular endothelial growth inhibitor (VEGI; TNFSF15) inhibits bone marrow-derived endothelial progenitor cell incorporation into Lewis lung carcinoma tumors. Angiogenesis.

[7] George AL, Bangalore-Prakash P, Rajoria S (2011). Endothelial progenitor cell biology in disease and tissue regeneration. J Hematol Oncol.

[8] Gazeau F, Wilhelm C (2010). Magnetic labeling, imaging and manipulation of endothelial progenitor cells using iron oxide nanoparticles. Future Med Chem.

[9] Yang JX, Tang WL, Wang XX (2010). Superparamagnetic iron oxide nanoparticles may affect endothelial progenitor cell migration ability and adhesion capacity. Cytotherapy.

[10] Wu H, Riha GM, Yang H (2005). Differentiation and proliferation of endothelial progenitor cells from canine peripheral blood mononuclear cells. J Surg Res.

[11] Stutchfield BM, Forbes SJ, Wigmore SJ (2010). Prospects for stem cell transplantation in the treatment of hepatic disease. Liver Transpl.

[12] Shabbir A, Zisa D, Suzuki G (2009). Heart failure therapy mediated by the trophic activities of bone marrow mesenchymal stem cells: a noninvasive therapeutic regimen. Am J Physiol Heart Circ Physiol.

[13] Zeng L, Hu Q, Wang X (2007). Bioenergetic and functional consequences of bone marrow-derived multipotent progenitor cell transplantation in hearts with postinfarction left ventricular remodeling. Circulation.

[14] Jickling G, Salam A, Mohammad A (2009). Circulating endothelial progenitor cells and age-related white matter changes. Stroke.

[15] Asahara T, Murohara J, Sullivan A (1997). Isolation of putative progenitor endothelial cells for angiogenesis. Science.

[16] Ding DC, Shyu WC, Lin SZ (2007). The role of endothelial progenitor cells in ischemic cerebral and heart diseases. Cell Transplant.

[17] Rotmans JI, Heyligers JM, Stroes ES (2006). Endothelial progenitor cell-seeded grafts: rash and risky. Can J Cardiol.

[18] Werner N, Kosiol S, Schieql T (2005). Circulating endothelial progenitor cells and cardiovascular outcomes. N Engl J Med.

[19] Moore MAS (2002). Putting the neo into neoangiogenesis. J Clin Invest.

[20] Mihail H, Wolfgang E, Peter CW (2003). Endothelial progenitor cells: mobilization, differentiation, and homing. Arterioscler Thromb Vasc Biol.

[21] Roberts N, Jahangiri M, Xu Q (2005). Progenitor cells in vascular disease. J Cell Mol Med.

[22] Werner N, Nickenig G (2006). Clinical and therapeutical implications of EPC biology in atherosclerosis. J Cell Mol Med.

[23] Modo M, Cash D, Mellodew K (2002). Tracking transplanted stem cell migration using bifunctional, contrast agent-enhanced, magnetic resonance imaging. Neuro Image.

[24] Hung SC, Deng WP, Yang WK (2005). Mesenchymal stem cell targeting of microscopic tumors and tumor stroma development monitored by noninvasive in vivo positron emission tomography imaging. Clin Cancer Res.

[25] Shichinohe H, Kuroda S, Lee JB (2004). In vivo tracking of bone marrow stromal cells transplanted into mice cerebral infarct by fluorescence optical imaging. Brain Res Brain Res Protoc.

[26] Weissleder R (1999). Molecular imaging: exploring the next frontier. Radiology.

[27] Daldrup-Link HE, Rudelius M, Oostendorp RA (2003). targeting of hematopoietic progenitor cells with MR contrast agents. Radiology.

[28] Togel F, Hu Z, Weiss K (2005). Administered mesenchymal stem cells protect against ischemic acute renal failure through differentiation-independent mechanisms. Am J Physiol Renal Physiol.

[29] Yocum GT, Wilson LB, Ashari P, Jordan EK, Frank JA, Arbab AS (2005). Effect of human stem cells labeled with ferumoxides-poly-L-lysine on hematologic and biochemical measurements in rats. Radiology.

[30] Jendelová P, Herynek V, Urdziková L (2005). Magnetic resonance tracking of human CD34^+^ progenitor cells separated by means of immunomagnetic selection and transplanted into injured rat brain. Cell Transplant.

[31] Weber A, Pedrosa I, Kawamoto A (2004). Magnetic resonance mapping of transplanted endothelial progenitor cells for therapeutic neovascularization in ischemic heart disease. Eur J Cardiothorac Surg.

[32] Crabbe A, Vandeputte C, Dresselaers T (2010). Effects of MRI contrast agents on the stem cell phenotype. Cell Transplant.

[33] Suh JS, Lee JY, Choi YS (2009). Efficient labeling of mesenchymal stem cells using cell permeable magnetic nanoparticles. Biochem Biophys Res Commun.

